# Morpho-Functional Architecture of the Golgi Complex of Neuroendocrine Cells

**DOI:** 10.3389/fendo.2013.00041

**Published:** 2013-03-28

**Authors:** Emma Martínez-Alonso, Mónica Tomás, José A. Martínez-Menárguez

**Affiliations:** ^1^Department of Cell Biology and Histology, Medical School, University of MurciaMurcia, Spain; ^2^Department of Human Anatomy and Embryology, Medical School, Valencia UniversityValencia, Spain

**Keywords:** golgi complex, neuroendocrine cells, morphology, transport vesicles, tubules, intra-golgi transport

## Abstract

In neuroendocrine cells, prohormones move from the endoplasmic reticulum to the Golgi complex (GC), where they are sorted and packed into secretory granules. The GC is considered the central station of the secretory pathway of proteins and lipids en route to their final destination. In most mammalian cells, it is formed by several stacks of cisternae connected by tubules, forming a continuous ribbon. This organelle shows an extraordinary structural and functional complexity, which is exacerbated by the fact that its architecture is cell type specific and also tuned by the functional status of the cell. It is, indeed, one the most beautiful cellular organelles and, for that reason, perhaps the most extensively photographed by electron microscopists. In recent decades, an exhaustive dissection of the molecular machinery involved in membrane traffic and other Golgi functions has been carried out. Concomitantly, detailed morphological studies have been performed, including 3D analysis by electron tomography, and the precise location of key proteins has been identified by immunoelectron microscopy. Despite all this effort, some basic aspects of Golgi functioning remain unsolved. For instance, the mode of intra-Golgi transport is not known, and two opposing theories (vesicular transport and cisternal maturation models) have polarized the field for many years. Neither of these theories explains all the experimental data so that new theories and combinations thereof have recently been proposed. Moreover, the specific role of the small vesicles and tubules which surround the stacks needs to be clarified. In this review, we summarize our current knowledge of the Golgi architecture in relation with its function and the mechanisms of intra-Golgi transport. Within the same framework, the characteristics of the GC of neuroendocrine cells are analyzed.

## Introduction

A century ago the Italian anatomist Camillo Golgi described a new organelle that nowadays bears his name, the Golgi apparatus or Golgi complex (GC) (Golgi, 1898). Using a silver impregnation method, the “black reaction,” he found a reticular structure in neurons that he called “apparato reticolare interno.” Due to the difficulties and variability inherent to the technique, it was not clear for decades whether this structure was anything more than an artifact. The electron microscope clearly demonstrated that, the GC is indeed a real organelle, which is composed of flattened cisternae surrounded by tubules and vesicles (Dalton and Felix, 1956). These first ultrastructural images obtained from ultrathin sections showed the exceptional complexity of the organelle and, consequently, high voltage electron microscopy and stereology were used to obtained 3D information (Rambourg et al., 1974). Ultrastructural immunocytochemical methods provide great impetus to the morpho-functional analysis of the GC through the precise location of key molecular components (Rabouille and Klumperman, 2005). Electron tomography has increased our knowledge of the 3D architecture of the GC (Ladinsky et al., 1999). Another advance has been the use of correlative light-electron microscopy, whereby cell organelles are visualized first by light microscopy in living cells transfected with fluorescent proteins, and then the same structures are identified under the electron microscope (Polishchuk et al., 2000; Mironov et al., 2008; van Rijnsoever et al., 2008). The combination of immunoelectron microscopy and electron tomography is a powerful approach for scrutinizing the secrets of this organelle (Zeuschner et al., 2006). In parallel to morphological approaches, biochemical and genetic analyses have described in detail the molecular machineries operating in the secretory/endocytic pathways.

The GC has two main functions. The first is the post-translational modification of proteins and lipids arriving from the endoplasmic reticulum (ER), mainly their glycosylation. The second function is the concentration, packing, and export of these modified products to the final destination in or outside of the cell. Thus, the GC is at the same time an efficient glycan factory and a post office. Helping to carry these functions is a surprisingly complex array of membranes equipped with an accurate machinery. Despite the large volume of incoming and outgoing traffic, it is able to maintain its architecture, although it is also flexible enough to disassemble and reassemble under certain conditions, such as mitosis. In neuroendocrine cells, prohormones are frequently glycosylated and proteolytically processed before being sorted into secretory granules (reviewed in Vázquez-Martínez et al., 2012). A summary of the current knowledge on the morphology of this organelle and early steps of the secretory pathway (i.e., ER-Golgi and intra-Golgi transport) is given below. Post-Golgi events, such as secretory granule formation, and other aspects of the Golgi functions are omitted in this description and can be found in excellent reviews elsewhere.

## Morphology of the Golgi Ribbon

**Figure 1:**
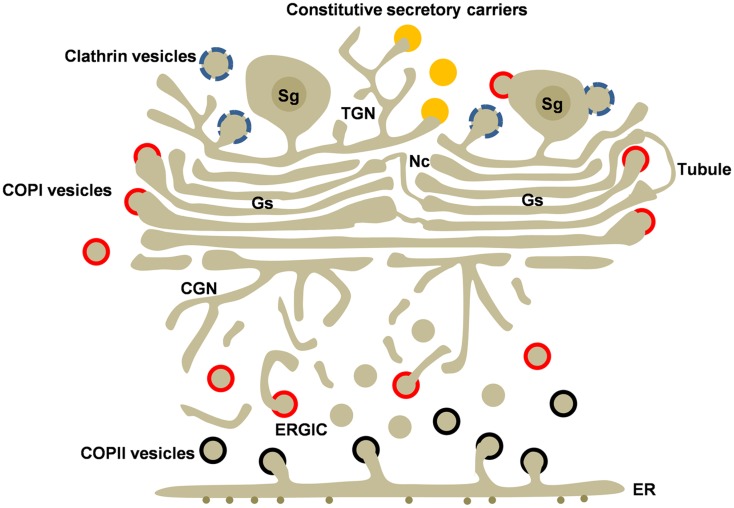
**Golgi structure and transport carriers in secretory cells**. The Golgi ribbon is formed by adjacent Golgi stacks (Gs) separated by non-compact regions (Nc) containing tubules and vesicles. Golgi stacks are connected to the cis and trans Golgi networks (CGN and TGN). Newly synthesized cargo leaves the endoplasmic reticulum (ER) by COPII-coated vesicles (black). COPI-coated (red) vesicles mediate recycling from the Golgi and the ERGIC. Transport carriers at the TGN include clathrin-coated vesicles (blue), regulated secretory granules (Sg), and the poorly understood constitutive secretory carriers (orange) Clathrin and COPI coats are also associated to secretory granules. Heterotypic and homotypic tubular connections between cisternae may be involved in anterograde and/or retrograde intra-Golgi transport.

**Figure 2:**
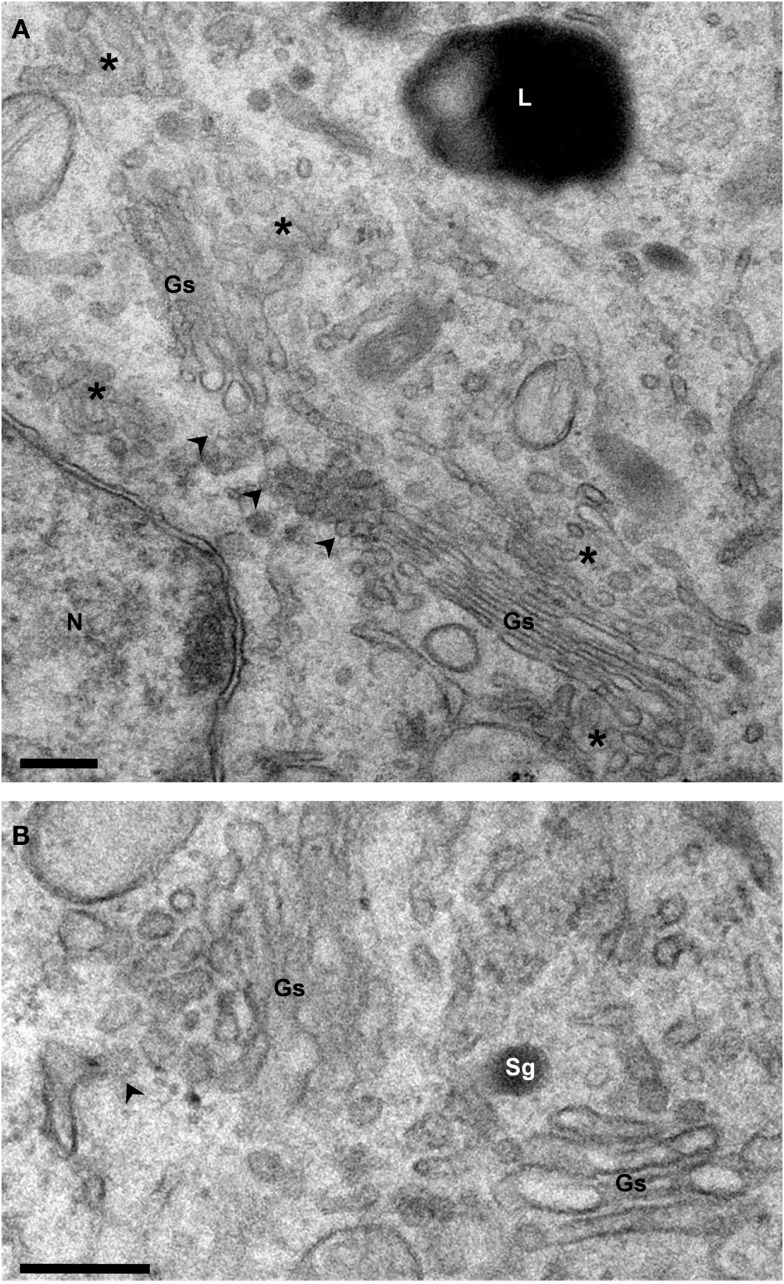
**Structure of the Golgi ribbon**. Electron micrographs of Epon-embedded PC12 cells. **(A)** The Golgi stacks (Gs) are surrounded by tubule-vesicular elements (asterisks). Arrowheads point to the non-compact area of the ribbon which connects the stacks laterally. **(B)** The cis and trans sides of the stacks are identified by the presence of ER exit sites (arrowhead) and secretory granules (Sg), respectively. Gs, Golgi stack; L, Lysosome; N, Nucleus. Bars, 200 nm.

**Figure 3:**
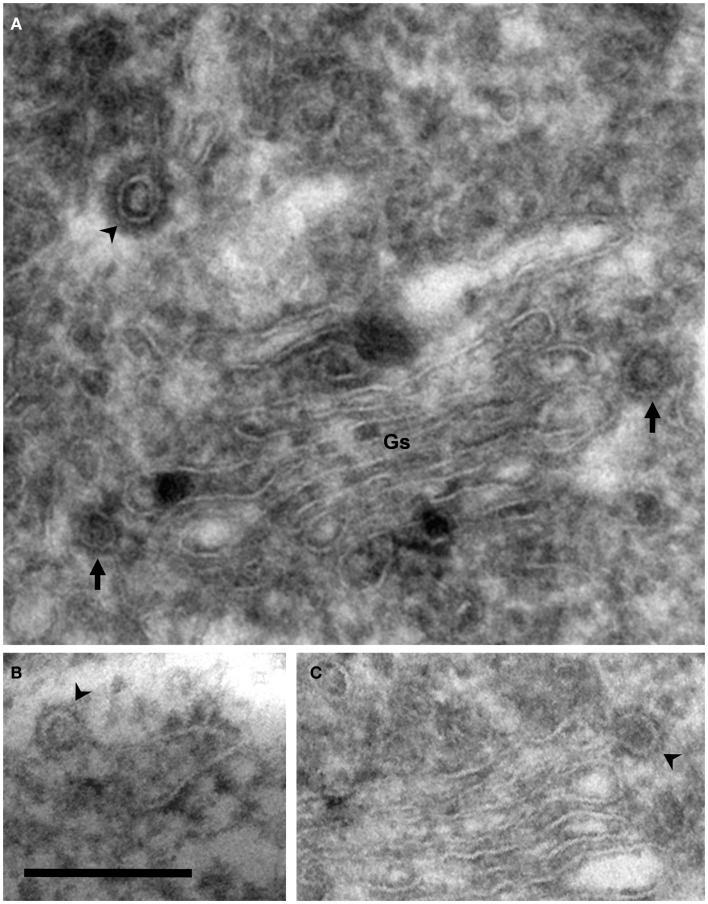
**Coated vesicles and buds**. Electron micrographs of cryosections of PC12 cells. Using this methodology, membranes appear negatively stained, whereas coats are identified as electron dense areas around vesicles and buds. **(A)** COP- (arrows) and clathrin-coated (arrowhead) vesicles are observed in the lateral and trans Golgi sides, respectively. Note the different thickness of these coats. **(B)** COPII-coated bud associated to the endoplasmic reticulum (arrowhead). **(C)** COPI-coated bud in the lateral rim of cisterna (arrowhead). COPII- and COPI-coated buds are identified by their locations because the thickness of these coats is identical. Bar, 200 nm.

In mammalian cells, this organelle consists of a pile of flat, disk-like membranes, the cisternae (Figures 1–3). This pile of cisternae is called the Golgi stack. The number of cisternae per stacks varies between different organisms but is characteristic of each species, usually numbering between 3 and 11 (Rambourg and Clermont, 1997). The diameter of the cisternae is also cell type-dependent, and is usually 0.5–1 μm (Weidman et al., 1993). The lumen of the cisternae is usually quite narrow (10–20 nm), allowing the interaction of the glycosylation enzymes present in its membranes and the cargo. Typically, cisternae are of uniform thickness in the central part but dilated near the lateral rims. In secretory cells, cisternae may show distensions filled with a material of low electron density, known as pro-secretory granules. These elements can be observed in the trans side alone (prolactin cells) or in all the cisternae, although, in the latter case, their size increases in the trans direction (Rambourg and Clermont, 1997). Cisternae may show small (fenestrae) and large holes, which are sometimes aligned to form wells (Ladinsky et al., 1999). Usually, such wells are filled with vesicles and exposed to both the cis and lateral sides of the stacks (Ladinsky et al., 1999). Fenestrations are less abundant in the medial cisternae of the stack and increase in both cis and trans directions.

Early histochemical and immunoelectron microscopical analysis demonstrated that the Golgi stack is polarized. Thus, based on the distribution of resident proteins, the Golgi stack can be divided into three regions: cis, medial, and trans. Glycosyltransferases, sugar nucleotide transporters, and many other Golgi proteins are preferentially found in one of these sub compartments. However, the resident proteins are not restricted to a few cisternae, but exhibit a gradient of concentration through out the cisternae of the stack, suggesting a state of dynamic equilibrium (Rabouille et al., 1995).

The GC of most mammalian cells is formed of several stacks that are laterally interconnected by tubules forming the Golgi ribbon (Rambourg et al., 1979; Rambourg and Clermont, 1997; Marsh et al., 2001) (Figures 1 and 2A). Thus, although it is not always apparent, the stacks observed in typical electron microscopic images of the Golgi area, belong to the same ribbon. Due to their appearance, the pile of cisternae and the lateral tubular network are called the compact and non-compact zones, respectively. Usually, the tubules connect cisternae located in the same positions in the respective stacks. However, heterotypic connections, even between the cis-most and trans-most cisternae of adjacent stacks, are abundant in some cell types (Rambourg and Clermont, 1997; Vivero-Salmerón et al., 2008). Frontal views of cisternae point to lateral networks of tubules emerging from the fenestrated rims (Weidman et al., 1993). Some of these tubular membranes fuse with the same cisterna, whereas others grow toward the cytoplasm. A single cisterna may have tubules oriented toward the cis and trans sides (Ladinsky et al., 1999). Many tubules, however, extend laterally and fuse with tubules from adjacent Golgi stacks, forming the tubular network that bridges adjacent stacks.

Usually, the ribbon is located close to the nucleus, around the microtubule organizing centers (MTOC), and the spatial configuration of the GC is closely related to the arrangement and orientation of the microtubules. The maintenance of the Golgi ribbon strongly depends on the microtubules and the action of motor proteins (Egea and Rios, 2008). Microtubule de-polymerizing agents such as nocodazole induce fragmentation of the ribbon into mini stacks (Storrie et al., 1998). In fact, the GC acts as a secondary MOTC. Golgi organization also depends on the actin cytoskeleton (Egea et al., 2006).

The structure of the ribbon is also supported by the so-called Golgi matrix, a ribosome-free area surrounding the cisternae (Xiang and Wang, 2011). This matrix can be visualized as small fibers connecting the cisternae (Mollenhauer and Morre, 1998) and also connecting the Golgi membranes and transport vesicles (Orci et al., 1998). The matrix is formed by structural proteins, some of them identified as auto-antigens and others isolated from detergent-insoluble salt-resistant Golgi fractions (Slusarewicz et al., 1994). These components include Golgi reassembly stacking proteins (GRASPs) and golgins (Xiang and Wang, 2011). These proteins are very dynamic and cycle between membrane-associated and a cytoplasmic forms.

The morphology of the GC (the number of cisternae per stack, the number of fenestrations, the complexity of associated tubule-vesicular elements, etc.) is cell type specific and depends on the activity of the cell. The level of cargo reaching the GC is an important factor in Golgi appearance. In general, when the input of cargo is low, the GC decreases in size and becomes larger when the synthetic activity is stimulated (Clermont et al., 1993; Taylor et al., 1997; Aridor et al., 1999; Glick, 2000). The relationship between cell activity and Golgi organization was clearly shown in prolactin cells. When the activity of these cells is reduced by removing the litters from lactating rats, the number of cisternal fenestrations and peri-Golgi vesicles increases concomitantly with a reduction in the number of Golgi tubules and mature secretory granules (Rambourg et al., 1993).

## Cis and Trans Golgi Network

The Golgi stack is flanked by two tubule-vesicular networks located at the cis and trans sides, which represent the entry and exit sides of the stack, respectively (Figure 1). At the cis-side, the cis-Golgi network (CGN) is involved in ER-Golgi transport. At the trans side, the trans Golgi network (TGN) receives and packs proteins and lipids that have traversed the stack and deliver them to their final destinations.

The CGN is formed of tubules connected to the first Golgi cisterna (Sesso et al., 1994; Rambourg and Clermont, 1997). This element is well developed in some cell types such as spermatids (Vivero-Salmerón et al., 2008) but less so in others, such as prolactin cells (Rambourg and Clermont, 1997). In early electron microscopical studies, these tubules were selectively identified by using reducing osmic for prolonged times. The functional relationship of this tubular network connected to the stack (the CGN) and ER-derived pre-Golgi elements [intermediate compartment (IC), see below] remains to be established.

Trans Golgi network is involved in the final steps of protein glycosylation and maturation and in the sorting of products to the apical and basolateral plasma membranes, early and late endosomes, and secretory granules (Griffiths and Simons, 1986; Keller and Simons, 1997; De Matteis and Luini, 2008). In neuroendocrine cells, the secretory proteins are concentrated in secretory granules that can be rapidly released after stimulation (Kelly, 1985). This regulated secretory pathway co-exists with the constitutive secretory pathway that is common to all cell types (Arvan and Castle, 1998). The sorting process can take place in the TGN (sorting-for-entry) or in immature secretory granules (sorting-by-retention) (Borgonovo et al., 2006). Different carriers and associated molecular machineries may be used for different routes (Traub and Kornfeld, 1997). Structurally, the TGN is formed of a large tubular network in continuity with the trans-most cisterna of the Golgi stack (Griffiths et al., 1985; Clermont et al., 1995). This is not always evident and, in some cell types, TGN can be found some distance from the stack (Clermont et al., 1995). The TGN can vary significantly in both size and composition, depending on the amount and type of cargo, and is reduced or absent in cells producing secretory granules in contrast with cells with an extensive lysosomal system (Clermont et al., 1995).

## Vesicles

The Golgi stack is surrounded by a high number of 50–100 nm vesicles, the smallest ones at the cis-side and lateral rims, and the largest ones at the trans side (Marsh et al., 2001). Many of these vesicles have a coat, an electron dense proteinaceous layer on the cytoplasmic leaflets of their membranes (Figure 3). These coats are also found in certain areas of the secretory/endocytic compartments, which represent forming vesicles. Three types of coat complex (COPI, COPII, and clathrin) have been identified and characterized. COPI- and COPII-coated vesicles are almost identical under the electron microscope. The overall size and coat thickness are 50–60 and 10 nm, respectively. COPII-coated buds are restricted to the ER, while COPI-coated buds are found in pre-Golgi and Golgi membranes (Griffiths et al., 1985; Oprins et al., 1993; Orci et al., 1997; Martinez-Menárguez et al., 2001; Rabouille and Klumperman, 2005). The trans-most cisternae of the Golgi and the TGN contains another type of coat, the clathrin coat (Pearse and Robinson, 1990) which is also found in the plasma membrane and endosomes. Clathrin-coated vesicles are unambiguously identified by their size (100 nm) and the thickness of the coat (18 nm) (Orci et al., 1984; Heuser and Kirchhausen, 1985; Kirchhausen et al., 1986; Oprins et al., 1993; Ladinsky et al., 2002). Interestingly, clathrin and COPI coats are also observed in forming secretory granules (Martínez-Menárguez et al., 1999).

Vesicles represent the best known type of transport intermediate. A huge amount of information has been accumulated on the molecular machinery involved in regulation of intercompartmental transport in the secretory pathway. Most data refer to vesicles as transport carriers but it can be assumed that the same or similar mechanisms operate in other carriers. While the formation of vesicles and the selection of cargo depend on the coat machinery, the specific targeting and fusion of the carriers with the target membranes depend on tethering factors, Rab and SNARE (soluble *N*-ethylmaleimide-sensitive factor attachment protein receptors) proteins, and other accessory proteins (Bonifacino and Glick, 2004).

SNARE proteins are involved in docking and the specific fusion of transport intermediates with the target membranes (Bonifacino and Glick, 2004; Hong, 2005; Jahn and Scheller, 2006). The SNAREs associated with vesicles (or other transport intermediates) and target membranes have been named v- and t-SNARE, respectively. SNAREs have also been divided into R- and Q-SNAREs, according to the central residue (R/Gln or Q/Arg, respectively) of the SNARE domain, a conserved region of 60–70 residues found in all members of this family. Commonly, v-SNARE and t-SNARE are R-SNARE and Q-SNARE, respectively. Interaction between one v-SNARE and two/three t-SNAREs induces the formation of the trans-SNARE complex, which catalyzes the fusion of the membranes. After fusion, a cis-SNARE complex is formed in the target membrane, which is later disassembled by the action of the cytosolic proteins α-SNAP (soluble *N*-ethylmaleimide-sensitive factor attachment protein) and the ATPase NSF (*N*-ethylmaleimide-sensitive factor). Now, v-SNARE can be transported back to the donor compartment to be reused. SNARE proteins are sufficient to drive membrane fusion so that they are considered the minimal membrane fusion machinery. Two SNARE complexes have been implicated in intra-Golgi transport (Malsam and Söllner, 2011). One complex is formed of v-SNARE GS15 and the t-SNAREs syntaxin5, GOS28, and Ykt6, and is involved in COPI-dependent intra-Golgi transport. The second complex is formed of v-SNARE rBet1 and the t-SNAREs syntaxin 5, membrin (GS27), ERS24/Sec22. This second complex has also been implicated in ER-to-Golgi and intra-Golgi transport (Volchuk et al., 2000). SNARE complex assembly is regulated by SM (Sec1/Munc18) proteins, a family of cytosolic proteins. Sly1 I is the only member of this family operating in the ER-Golgi area (Laufman et al., 2009), whereas Munc18-1 is involved in the exocytosis of dense-core granules in neuroendocrine cells (Burgoyne et al., 2009).

Rab proteins are a family of small GTPases that regulate membrane transport by recruiting effectors, including sorting adaptors, tethering factors, kinases, phosphatases, and motor proteins (Jahn and Scheller, 2006; Stenmark, 2009). Rab proteins switch between an active form (GTP-bound) and a cytosolic inactive form (GDP-bound). They have been implicated in vesicle budding, uncoating, mobility, and transport. Rab proteins in the GTP-bound form are reversibly associated with membranes by geranylgeranyl groups. The replacement of GDP by GTP is facilitated by guanine nucleotide exchange factors (GEFs), and their low intrinsic GTPase activity is enhanced by GTPase-activating proteins (GAP). Rab protein is a large family, including more than 60 members in humans, which are specifically associated with distinct compartments and transport events (Stenmark, 2009). Interestingly, the specific membrane recruitment of Rabs has recently been demonstrated to depend on the activity of GEFs (Blümer et al., 2013). The Golgi-associated Rab family playing a key role in Golgi maintenance and functioning includes Rab1, Rab2, Rab6, Rab33B, Rab18, and Rab43 (Liu and Storrie, 2012). Rab3 (A–D), Rab11, Rab18, Rab26, Rab27 (A,B), and Rab37 are involved in regulated secretion (Fukuda, 2008; Stenmark, 2009). Rab3A, Rab11, Rab18, and Rab27A regulate exocytosis in neuroendocrine cells by interacting directly with secretory vesicles (Vázquez-Martínez and Malagón, 2011). A few GEFs and GAPs for secretory Rabs have been identified. The specific role of secretory Rabs in the formation and maturation of the secretory vesicles, and their docking and fusion with the plasma membrane, remains controversial.

Tethering factors are a group of membrane-associated proteins or multi-subunit complexes that link transport vesicles with the target membranes to ensure correct docking and fusion. In addition to tethering, they play a role in Golgi stacking and form the Golgi matrix. They have been classified into three classes: oligomeric complexes that work as Rab effectors and bind SNARE, oligomeric complexes that function as GEFs for Rab proteins and, finally, coiled-coil tethers (Sztul and Lupashin, 2009). Golgi-associated members of the last group are called golgins. The golgin family includes p115, a protein believed to be involved in tethering COPII vesicles to pre-Golgi membranes, transport from these elements to the cis-Golgi and intra-Golgi transport. GM130 is another coiled-coil protein associated with the cis-Golgi. GM130 and p115 are also components of the Golgi matrix. Apart from their role as tethers, they are involved, together with other members of the golgin and GRASP families such as giantin and GRASP65, in maintaining the stacked morphology of the cisterna and the Golgi ribbon (De Matteis et al., 2008). NECC1 (neuroendocrine long coiled-coil protein 1) is a new component of this family, which is mainly present in neuroendocrine tissues (Cruz-Garcia et al., 2007). NECC1 is the first long coiled-coil protein described that has a role as a negative modulator of the regulated secretion in neuroendocrine cells (Cruz-García et al., 2012). Dsl1, conserved oligomeric Golgi (COG), and transport protein particle (TRAPP) are multi-subunit complexes associated with the GC (Sztul and Lupashin, 2009). Dsl1 is a three unit complex involved in Golgi-to-ER transport, where it acts to tether COPI vesicles. The COG complex formed of eight subunits (Cog1–8) found at the cis and medial Golgi cisternae is believed to be involved in transport to the Golgi and the intra-Golgi recycling of Golgi resident proteins (Miller and Ungar, 2012). TRAPP is another multi-subunit complex that works as tethering factor (Sacher et al., 2008). At least two TRAPP complexes exist in mammals. TRAPPI tethers COPII-coated vesicles and mediates ER-to-Golgi transport (Barrowman et al., 2010). TRAPII has been involved in intra-Golgi transport, post-Golgi traffic, endosome-to-Golgi, and autophagy (Yu and Liang, 2012). They work as GEF for Rab1 and the activation of this GTPase might recruit other tethers (Sztul and Lupashin, 2009).

Some lipidic species, such as diacylglycerol, phosphatidic acid and lysophosphatidic acid, and enzymes associated with their metabolism play a key role in carrier formation by regulating the curvature of membranes. Diacylglycerol has been implicated in the formation of post-Golgi carriers and is necessary to recruit protein kinase D, a regulator of the fission of transport carriers (Bard and Malhotra, 2006). Diacylglycerol has also seen to be involved in Golgi-to-ER retrograde transport mediated by tubules (Fernández-Ulibarri et al., 2007). Phosphatidic acid, which is generated by phospholipase D2, is involved in COPI vesicle formation (Yang et al., 2008). This phospholipid is necessary to maintain the structure of the GC and secretion in neuroendocrine cells (Siddhanta et al., 2000). Lysophosphatidic acid generated by the enzyme phospholipase A2 has been involved in the retrograde transport mediated by tubules (de Figueiredo et al., 2000; Brown et al., 2003). This enzyme is involved in the formation of tubular continuities between cisternae (San Pietro et al., 2009) and tubular transport intermediates at the TGN (Schmidt et al., 2010). Interestingly, two lipid-modifying enzymes, lysophosphatidic acid acyltransferase γ and phospholipase A2-α, which promote or inhibit COPI fission, respectively, work together, regulating the morphology of Golgi carriers (vesicles vs. tubules) (Yang et al., 2011).

## The Golgi Complex of Neuroendocrine Cells

The hypothalamus-hypophysis system is the most important and well-known neuroendocrine system. Important clues on Golgi functioning have been obtained by studying the GC of the pituitary gland. In addition, neuroendocrine cell lines, such as corticotropic tumor AtT20, pheochromocytoma PC12, and frog melanotrope cells, provide important clues as regards secretory granule formation and regulation (Morvan and Tooze, 2008). Thus, the discovery that immature secretory granules originated from Golgi cisternae was made in mammotroph cells (Farquhar, 1961). In the same cell type it was also found that that there is a step during which secretory material is condensed in the GC (Smith and Farquhar, 1966). AtT20 cells have been used to show that there are different routes from the GC to the plasma membrane (Gumbiner and Kelly, 1982). However, detailed morphological studies of the Golgi ribbon of neuroendocrine cells, with some exceptions, are scarce. The morphology of the GC and the formation of secretory granules in prolactin cells were described in early microscopic studies. In these cells, the Golgi ribbon forms a hollow sphere in the perinuclear area. The stacks of prolactin cells have four to five (mostly flattened) cisternae and show a reduced CGN and TGN (Rambourg and Clermont, 1997). The trans cisternae show distensions that are gradually transformed into tubular progranules at the trans face and are finally condensed into compact polymorphous granules (Clermont et al., 1993). As indicated above, the morphology of this compartment is strongly dependent on cell activity (Rambourg et al., 1993). Many other cells of the anterior pituitary gland have not been extensively studied, although some of these cell types have a spherical GC, with cis and trans side representing the outer and inner part of this sphere, respectively (Watanabe et al., 2012).

## ER-to-Golgi Transport

Newly synthesized proteins and lipids in general, and prohormones in neuroendocrine cells, exit the ER in specific places of this compartment, called ER exit sites (ERES). These places are formed by tubular buds of different length containing a COPII coat (Sesso et al., 1994; Bannykh et al., 1996; Zeuschner et al., 2006), which assists in the deformation of ER membranes into vesicles containing membrane and soluble cargo *en route* to the GC (Barlowe et al., 1994). The COPII coat complex is formed of five soluble proteins: Sec23, Sec24, Sec13, Sec31, and Sar1 (Barlowe et al., 1994; Bickford et al., 2004). Formation of the COPII coat begins with the recruitment of the GTPase Sar1 in ER membranes. This binding depends on the activation of this GTPase by Sec12, a GEF present in ER membranes (Jensen and Schekman, 2011). During this process some proteins are selectively recruited into COPII vesicles (Barlowe, 1998, 2003), whereas others enter unspecifically, a process known as bulk flow (Martínez-Menárguez et al., 1999). Sorting of the transmembrane cargo depends on Sec24 (Mancias and Goldberg, 2008). The soluble cargo present in the lumen of the ER binds to cargo receptors, such as ER-Golgi IC (ERGIC)-53, p24, and the Erv families (Szul and Sztul, 2011). These cargo receptors cycle between the ER and Golgi and also are included in recycling COPI vesicles. The p24 family comprises 8–10 isoforms and a subset of these proteins is up-regulated, together with proopiomelanocortin, after the activation of neuroendocrine frog melanotrope cells (Strating et al., 2011). The size of COPII vesicles is regulated by ubiquitination of Sec31, allowing the formation of large COPII vesicles (Jin et al., 2012). After formation, COPII vesicles are quickly uncoated and fuse to each other to form the so-called ERGIC, IC, or vesicular-tubular clusters (VTCs), the first of these being the most used (Hauri and Schweizer, 1992; Farquhar and Hauri, 1997). This compartment was initially identified as tubule-vesicular membranes in which the cargo accumulates when cells are cultured at low temperatures (15°C) (Saraste and Kuismanen, 1984; Schweizer et al., 1990). This compartment is located close to the GC and is also distributed throughout the cell (Lotti et al., 1992; Klumperman et al., 1998), and is associated to ERES (Bannykh et al., 1996). ERGIC is formed by vesicles and tubules, sometimes branched (Bannykh et al., 1996). Although adjacent to the ERES, ERGIC is an independent compartment and there is no continuity between them (Bannykh et al., 1998). ERGIC membranes do not have COPII coats but another type of coat complex, the COPI coat, which is involved in retrograde transport (see below). Thus, the presence of these coats can be used to discriminate between these closely related compartments. Whether the ERGIC is a stable compartment or a transitory element moving toward the GC is still a matter of debate (Ben-Tekaya et al., 2005). The fact that all the proteins associated with this compartment cycle between the ER and Golgi argues against the idea that it is a stable compartment. ERGIC-53, a type I transmembrane protein of the lectin family, is the prototypical marker of this compartment (Zhang et al., 2009). However, *in vivo* experiments showed that ERGIC-53 is located in long-lived stationary elements connected by highly mobile elements (Ben-Tekaya et al., 2005), supporting the view that ERGIC is a stable compartment. ERGIC elements or ERGIC-derived carriers move to the Golgi area along the microtubule track guided by dynein motors, where they may fuse to each other to form the CGN or, conversely, fuse with a pre-existing cisterna (Presley et al., 1997).

## Golgi-to-ER Transport

Organelle identity is determined by its composition, and its functional integrity is due to its ability to maintain this composition despite the continuous traffic between compartments. An important mechanism involved in this process is retrograde transport. In the early secretory pathway, ERGIC is the first place where this process occurs. Here, the presence of COPI coats ensure the recycling of proteins to the ER while anterograde cargo is separated and concentrated (Scales et al., 1997; Klumperman et al., 1998; Martínez-Menárguez et al., 1999; Shima et al., 1999; Stephens et al., 2000). The function of the COPI coats in the Golgi-to-ER retrograde transport of soluble and membrane proteins has been convincingly demonstrated (Letourneur et al., 1994). However, there are retrograde routes to the ER independent of COPI (Girod et al., 1999). It is also possible that a population of COPI vesicles is involved in anterograde transport across the Golgi stack (Orci et al., 1997).

COPI-coated vesicles are found in peripheral and central ERGIC elements, at cis and lateral Golgi sides (Oprins et al., 1993) and, occasionally, at the trans Golgi side/TGN (Martínez-Menárguez et al., 1996). COPI-coated buds are observed at the lateral rims of cisternae, decreasing in number in a cis to trans direction (Ladinsky et al., 1999). COPI coats are formed of seven subunits (α, β, β’, γ, δ, ε, ζ-COP) assembled in the cytosol, the coatomer, and small GTPase ADP-ribosylation factors (Arf1). Arf1 belong to the Arf family, which is made up of six members in mammals (D’Souza-Schorey and Chavrier, 2006). Arfs 1–5 have been described at the ER-Golgi interface, where they may have redundant functions. Besides, it has been suggested that different pairs of Arfs may be necessary for each transport step (Volpicelli-Daley et al., 2005). Arf1 and 4 and Arfs 3–5 are associated to the cis and trans Golgi sides, respectively (Donaldson and Jackson, 2011). Arf 4 and 5 interact with calcium-dependent activator for secretion (CASP), regulating the formation of neuroendocrine secretory granules (Sadakata et al., 2010). Arf1-GDP is recruited to membranes by p23, a member of the p24 family (Gommel et al., 2001), and, once there, it is activated by a GEF. GTP-bound Arf1 is able to recruit coatomer *en bloc* to membranes. At the ERGIC and Golgi membranes, GBF1 (Golgi-associated BFA-resistant protein) is the major GEF for ARF1 during COPI vesicle formation and is the target of the drug brefeldin A (Kawamoto et al., 2002; Garcia-Mata et al., 2003), while Big1 and Big2 (BFA-inhibited GEF) are described as GEFs for Arf1 in the TGN and endosomes (Ishizaki et al., 2008). ArfGAPs stimulate the GTP hydrolysis of Arf, which has low intrinsic GTPase activity (Inoue and Randazzo, 2007). It has been postulated that this reaction triggers uncoating (Tanigawa et al., 1993); however, ArfGAP1 has also been described as a coatomer component and also as a sensor of membrane curvature, so the role of this protein is under discussion (Shiba and Randazzo, 2012). ArfGAP1, phosphatidic acid generated by phospholipase D and BARS (brefeldin A-ribosylated substrate) have been implicated in COPI vesicle fission (Yang et al., 2008).

Many transmembrane proteins transported into COPI vesicles bear di-lysine motifs at their C-terminus (Letourneur et al., 1994), including ERGIC-53 (Schindler et al., 1993). The coatomer subunits, α- and β’-COP, directly bind this signal (Jackson et al., 2012). The p24 family of proteins is recruited by direct interaction of their cytoplasmic tail, which contains phenylalanine- and di-lysine-based signals, with γ-COP (Nickel et al., 1997; Béthune et al., 2006). Not all cargo incorporated in COPI vesicles has this sorting signal so that the cargo may require adaptors. This is the case with glycosylation enzymes (Popoff et al., 2011). One example of an adaptor for soluble proteins is the KDEL receptor, a transmembrane protein mostly present in the cis-Golgi and ERGIC, which interacts with the coatomer through a di-lysine motif in the cytosolic tail, whereas the luminal part interacts with soluble proteins bearing a K-D-E-L sequence found in many ER-resident proteins (Semenza et al., 1990; Majoul et al., 2001). In this way, soluble proteins that have escaped from the ER are retrieved.

As indicated above, it is possible that there are several subpopulations of COPI vesicles with different compositions and locations, each carrying out its specific functions (Moelleken et al., 2007). Indeed, γ and ζ-COP present two isoforms localized differently along the GC, enabling the existence of four types of coatomer, which may act in different routes and types of cargo (Popoff et al., 2011). Thus, this coat conforms versatile vesicles that may play different roles at the ER-Golgi interface (Orci et al., 1997; Shima et al., 1999; Malsam et al., 2005).

## Intra-Golgi Transport

Once cargo has reached the GC, the manner in which it moves through the stack remains controversial, and two classical models have been proposed: vesicular transport and cisternal maturation. The first proposes that Golgi cisternae are static so that the cargo must use vesicles to move. The second model proposes that cisternae are dynamic structures that move from the cis to trans Golgi sides. Thus, according to this model, the entire cisterna is the carrier. The postulated roles of COPI vesicles in vesicular and cisternal maturation models are completely different, since they are regarded as being responsible for anterograde and retrograde transport, respectively.

Launched by Palade (1975), the vesicular transport model postulates that cisternae are stable compartments. Thus, cargo must leave one cisterna and move to an adjacent one in order to advance through the stack. This process is mediated by COPI vesicles. For decades this model was widely accepted since it was based on new experimental data, especially the analysis of cell-free systems and immunomicroscopical studies (Rothman and Fine, 1980; Orci et al., 1986; Barlowe et al., 1994). It provided a good explanation for the well-known compartmentalization of the Golgi resident enzymes (Roth and Berger, 1982; Dunphy et al., 1985). Besides, it was strongly supported by the discovery of COPI and COPII vesicles which, it was postulated, act sequentially in the early secretory pathway (Rothman and Wieland, 1996). It was clearly demonstrated that COPII vesicles are involved in ER exit (Barlowe et al., 1994), while COPI were identified in *in vitro* assays as being responsible for anterograde transport between cisternae (Orci et al., 1986). However, this model was questioned when a clear role for COPI vesicles in retrograde transport was demonstrated (Cosson and Letourneur, 1994; Letourneur et al., 1994). Even so, the model remained in force even when two types of COPI vesicle were seen to be involved in anterograde and retrograde transport in the Golgi stack (Orci et al., 1997). The weakest point of this model is that it does not explain how large cargo is transported.

A cisternal maturation model was postulated by Grasse (1957) based on early electron microscopy observations, but it was not until the early 1990s that it was re-considered (Bonfanti et al., 1998; Glick and Malhotra, 1998). According to this model, cisternae are formed at the cis-side of the GC by fusion of ER-derived membranes and then these newly formed cisternae move from the cis to the trans side. This model fits very well with the observation that the Golgi resident enzymes are not strictly compartmentalized through the Golgi stack (Nilsson et al., 1993; Rabouille et al., 1995). Furthermore, this model is able to explain the transport of large cargo, such as procollagen (300 nm rigid rod) (Bonfanti et al., 1998) or algal scales (up to 1.5–2 μm) (Melkonian et al., 1991), which do not fit within 50–60 nm vesicles. During cisternal progression, Golgi resident enzymes must be packed into COPI vesicles and transported backwards. Thus, Golgi enzymes are not lost but recycled in a trans to cis direction, maintaining the polarity of the organelle (Glick et al., 1997). Immunocytochemical (Martinez-Menárguez et al., 2001; Mironov et al., 2001) and proteomic (Gilchrist et al., 2006) analyses of these vesicles showed that they are mostly devoid of anterograde transport markers but enriched in glycosylation enzymes. However, other studies on COPI vesicle composition provided conflicting results. Thus, the role of COPI vesicles remains controversial (Cosson et al., 2002). Life cell imaging studies in yeast involving direct real time visualization of cisternal maturation strongly support this model (Losev et al., 2006; Matsuura-Tokita et al., 2006).

Since neither of these models explains all the experimental data, a combination of the same models (dual model) as well as new models (rapid partitioning, kiss-and-run) have been proposed (reviewed in Glick and Luini, 2011). The cisternal maturation model seems to be more efficient, given that it seems easier to transport anterograde cargo using a large carrier (the cisterna) than to use many small vesicles between adjacent cisternae and repeat this process several times until reaching the TGN. However, these models are not mutually exclusive and a combination of both might serve (Pelham and Rothman, 2000). Cisternae may move slowly, whereas vesicles transport anterograde and retrograde cargo more rapidly. Large molecules may use the cisternal maturation mechanism, while small molecules can be transported using vesicular transport (Orci et al., 2000; Pelham and Rothman, 2000). In addition, other mechanisms might operate in this transport step as several new models postulate. According to the rapid partitioning model, the GC operates as a single compartment but contains processing and export domains of differing lipid composition, which allow the specific retention of resident enzymes and cargo proteins (Patterson et al., 2008). The cargo associates with these domains and then leaves the compartment from every level. However, this model has difficulty in explaining many previous observations including the well-known polarized distribution of glycosylation processing enzymes, the progression of procollagen and other cargo across the stack or the role of COPI vesicles. The kiss-and-run model, meanwhile, proposes that two cisternae may fuse to each other through narrow tubules, connecting their lumens transiently, and allowing the transit of anterograde and retrograde cargo before disconnection (Mironov and Beznoussenko, 2012). Given that specific retrograde transport is not necessary in this model, COPI might be involved in the fission process. It is also not clear how all the experimental data available concerning the role of COPI coats in retrograde transport fit this model.

Despite the abundance and development of Golgi-associated tubules, as described above, most models of intra-Golgi transport do not include a role for these elements. Tubules may act as intermediate transport carriers, alone or with vesicles, and in anterograde and retrograde transport (Griffiths, 2000; Mironov et al., 2003; Marsh et al., 2004; Trucco et al., 2004; Martínez-Alonso et al., 2005, 2007; Vivero-Salmerón et al., 2008). In fact, it has recently been found that a similar mechanism involving COPI and lipid-modifying enzymes may regulate the formation of both types of carrier in the GC (Yang et al., 2011). With some exceptions, intercisternal tubular connections are scarce in control cells but increase when secretory activity is stimulated (Marsh et al., 2004; Trucco et al., 2004), supporting the view that tubules may be involved in anterograde transport when there is an excess of cargo. In fact, it has been postulated that all secretory compartments are connected by tubules and the cargo moves along this pathway like food through the gut (Griffiths, 2000). Supporting this idea, although unusual, a Golgi stack formed by a single cisterna arranged helically and direct connections between the Golgi cisternae and the ER have been described (Tanaka et al., 1986). In order to clarify the role of tubules, it is first necessary to determine their composition, which is a difficult task. The number of tubules can be increased by the use of the fungal drug brefeldin A. Nowadays it is well accepted that brefeldin A-induced tubules are involved in Golgi-to-ER transport. Although this system is artificial, it is believed that brefeldin A intensifies a process that occurs naturally (Lippincott-Schwartz et al., 1990). Tubules can also be enhanced by lowering the temperature (Martínez-Alonso et al., 2005, 2007). These tubules exclude anterograde and retrograde cargo but recruit Golgi resident enzymes and a specific set of Rab and SNARE proteins involved in intra-Golgi transport (Martínez-Alonso et al., 2005, 2007). Thus, these induced tubules may be indicative of the recycling mechanisms of Golgi enzymes postulated by the cisternal maturation model.

## Concluding Remarks

The GC has fascinated scientists for more than a century. Although it is without doubt the most photographed cell structure, it still retains most of its mystery. What is the reason for its beautiful architecture? Why does it form a ribbon in most cells? What are that the functions of the tubular networks? Do vesicles and tubules have specific functions? Is there a single mode of intra-Golgi transport? Do COPI vesicles take part in anterograde transport? Is the ERGIC a real compartment? These and many other questions remain unanswered, which, in itself, is surprising, given the intense research and the large number of research groups working in the field. It is to be hoped that new approaches and research models will help fill the gaps in our knowledge of this beautiful organelle.

## Conflict of Interest Statement

The authors declare that the research was conducted in the absence of any commercial or financial relationships that could be construed as a potential conflict of interest.
